# Microvesicle Involvement in Shiga Toxin-Associated Infection

**DOI:** 10.3390/toxins9110376

**Published:** 2017-11-19

**Authors:** Annie Villysson, Ashmita Tontanahal, Diana Karpman

**Affiliations:** Department of Pediatrics, Clinical Sciences Lund, Lund University, 22184 Lund, Sweden; annie.villysson@med.lu.se (A.V.); ashmita.tontanahal@med.lu.se (A.T.)

**Keywords:** Shiga toxin, hemolytic uremic syndrome, enterohemorrhagic *Escherichia coli*, microvesicles, kidney

## Abstract

Shiga toxin is the main virulence factor of enterohemorrhagic *Escherichia coli*, a non-invasive pathogen that releases virulence factors in the intestine, causing hemorrhagic colitis and, in severe cases, hemolytic uremic syndrome (HUS). HUS manifests with acute renal failure, hemolytic anemia and thrombocytopenia. Shiga toxin induces endothelial cell damage leading to platelet deposition in thrombi within the microvasculature and the development of thrombotic microangiopathy, mostly affecting the kidney. Red blood cells are destroyed in the occlusive capillary lesions. This review focuses on the importance of microvesicles shed from blood cells and their participation in the prothrombotic lesion, in hemolysis and in the transfer of toxin from the circulation into the kidney. Shiga toxin binds to blood cells and may undergo endocytosis and be released within microvesicles. Microvesicles normally contribute to intracellular communication and remove unwanted components from cells. Many microvesicles are prothrombotic as they are tissue factor- and phosphatidylserine-positive. Shiga toxin induces complement-mediated hemolysis and the release of complement-coated red blood cell-derived microvesicles. Toxin was demonstrated within blood cell-derived microvesicles that transported it to renal cells, where microvesicles were taken up and released their contents. Microvesicles are thereby involved in all cardinal aspects of Shiga toxin-associated HUS, thrombosis, hemolysis and renal failure.

## 1. Introduction

Shiga toxin-producing *Escherichia coli* (STEC) or enterohemorrhagic *E. coli* (EHEC) may cause disease in humans manifesting with diarrhea, bloody diarrhea (hemorrhagic colitis) and, in approximately 15% of cases, the severe systemic complication of hemolytic uremic syndrome (HUS) [1]. HUS is characterized by the post-diarrheal acute onset of non-immune hemolytic anemia, thrombocytopenia and renal failure. The most common clinical EHEC isolate is *E. coli* O157:H7 [2], although many other non-O157 serotypes have been described, notably the *E. coli* O104:H4 serotype that caused a huge outbreak in 2011 [3]. EHEC is a non-invasive pathogen [4] that colonizes the intestine where it expresses and also releases virulence factors. Some of these allow adherence to the intestinal mucosa by forming attaching and effacing lesions leading to colonization [5], while flagella are associated with bacterial motility [6]. EHEC interaction with commensal strains and host hormones enhances colonization and virulence by a genetically determined phenomenon known as quorum sensing [7]. The major and unique virulence factor strongly associated with EHEC-induced morbidity is Shiga toxin [8]. In addition, EHEC possesses lipopolysaccharide (LPS) and other factors capable of activating the host response [9]. A prerequisite for the strain to cause systemic and target organ damage, such as renal failure or brain damage [10], is the ability of virulence factors to gain access to the bloodstream and thereby reach target organ cells.

Shiga toxin may be capable of binding to intestine epithelial cells and thereafter translocate [11,12,13]. The intestinal inflammatory response is multifactorial depending on the interaction between the toxin, other virulence factors, and the host response [9]. Shiga toxin-producing EHEC strains are diarrheogenic. The diarrhea may become bloody leading to hemorrhagic colitis. This form of intestinal injury appears to be specifically associated with Shiga toxin production, as demonstrated in a monkey model of Shigella infection [14]. The massive erosion of the intestinal mucosal lining allows virulence factors released from EHEC to gain access to the circulation. Once within the bloodstream most of the toxin does not circulate in free form [15,16] but rather bound to blood cells such as leukocytes [17] and platelets as well as aggregates between these cells [18]. Red blood cells are also capable of binding the toxin [19,20]. Blood cells are activated by toxin binding and, thereafter, shed microvesicles which are pro-inflammatory, pro-thrombotic [18], and, importantly, transport the toxin to its target organ [21]. This does not exclude other mechanisms of toxin transfer from blood cells to affected cells [22], but has been suggested to be one of the main mechanisms of toxin-induced systemic and targeted organ injury [1].

Microvesicles are a subtype of extracellular vesicles shed directly from the plasma membrane of cells upon activation, stress and apoptosis [23]. Microvesicles can originate from blood cells [24,25,26] as well as non-circulating organ-specific cells [27,28]. Vesicles may be enriched in components of the parent cells such as proteins, receptors, RNAs (mRNA and miRNA) and lipids, enabling them to interact with cells in their immediate vicinity and at a distance [29]. Vesicle release may also maintain cellular integrity by ridding the cell of harmful substances [30]. Increasing evidence suggests that microvesicles are key players in several diseases, including cancer [31], renal diseases [32], cardiovascular disease [33] and inflammatory diseases [34]. In these diseases, the number of circulating microvesicles is significantly increased, indicating a disruption in physiological processes. In Shiga toxin-associated disease, Shiga toxin-bearing microvesicles have been found in the circulation of EHEC-infected patients as well as within the kidney [21], enabling toxin evasion of the immune system and thereby protection of the toxin from degradation. This review will mainly focus on the functions of microvesicles, in general and in the context of bacterial infections, particularly with respect to Shiga toxin-associated infection.

## 2. Shiga Toxin

Shiga toxin, encoded by a bacteriophage, is released from bacteria in the gut, most probably during bacterial lysis [35]. Shiga toxin is a ribosomal-inactivating protein. It is an AB_5_ toxin composed of two subunits, an A-subunit and a pentrameric B-subunit, linked together by non-covalent bonds [36]. The A-subunit accounts for the enzymatic cytotoxic activity whereas the pentameric B-subunit binds to glycosphingolipid receptors mainly the globotriaosylceramide (Gb3) receptor [37,38] and, to a lesser extent, the Gb4 receptor [39]. The density of Gb3 in the cell membrane and its association with lipid rafts affect toxin binding [40].

After Shiga toxin binds to its glycolipid receptor it can be taken up by endocytosis. Various endocytic routes have been described involving formation of membrane microtubular structures mainly in a clathrin-independent manner but also by a clathrin-dependent mechanism [41,42,43,44], as recently reviewed [45]. Uptake in intestinal cells by macropinocytosis, in a Gb3-independent manner, has also been reported [46,47]. Once within a cell, Shiga toxin is ultimately destined to reach ribosomes in the cytosol [48]. Shiga toxin is transported in a retrograded manner from early endosomes to the trans-Golgi network and further to the endoplasmic reticulum. Within the endoplasmic reticulum the A subunit is cleaved by furin into the A1 and A2 subunits [49]. From the endoplasmic reticulum, Shiga toxin is transported out to the cytosol, accessing the ribosomes [50].

### 2.1. Cytotoxicity of Shiga Toxin

The enzymatically active A1 subunit of Shiga toxin exerts a cytotoxic effect by *N*-glycosidic cleavage of a specific adenine base from 28S rRNA [51] leading to inhibition of protein synthesis followed by cell death. Moreover, Shiga toxin activates apoptotic pathways [52,53], most probably by inducing ribosomal damage and further activation of mitogen-activated protein (MAP) kinase pathways, the so-called ribosomal stress response [54,55]. Shiga toxin has been shown to induce intestinal cell apoptosis [56] and renal cell apoptosis in vivo and in vitro [52].

### 2.2. Inflammatory Effects of Shiga Toxin and Lipopolysaccharide in the Intestine

In addition to its cytotoxic effects, Shiga toxin is capable of activating an inflammatory response in the intestine, in its interaction with blood cells and after binding to target organ cells [9]. These effects occur simultaneously with the cytotoxic effects and are associated with the release of a wide range of pro-inflammatory cytokines and chemokines. In vitro studies have shown that Shiga toxin can trigger neutrophil influx into the intestine by inducing the release of interleukin (IL)-8 and other C-X-C chemokines [57,58,59]. The interaction with peritoneal macrophages also led to release of pro-inflammatory cytokines such as tumor necrosis factor-alpha (TNF-α) and IL-6 [60]. LPS may also contribute to the inflammatory response in the intestine [60]. Studies in mice have indicated that the initial host response to *E. coli* O157:H7 LPS is essential for bacterial elimination from the gut, thus mice lacking an adequate response to LPS were subject to more severe disease [61,62].

### 2.3. Interactions between Shiga Toxin and Blood Cells

During HUS, elevated neutrophil counts and decreased platelet counts suggest a worse prognosis [63]. Shiga toxin may circulate bound to neutrophils in vivo [17,64], and aggregates between platelets and neutrophils [18]. Both Shiga toxin and LPS induce the formation of these cell aggregates in which neutrophils are activated. Thus, the toxin may induce neutrophil activation and degranulation [9]. Similarly, the toxin may bind to monocytes [65] and has been detected on monocytes or platelet-monocyte aggregates in patient samples [18]. Shiga toxin induces the release of pro-inflammatory cytokines from monocytes including IL-6, IL-8, TNF-α, IL-1β and RANTES [65,66], as well as the expression of pro-thrombotic tissue factor activating the extrinsic pathway of coagulation leading to thrombin generation and blood clotting [18,67].

Platelets are activated during HUS and deposit in microthrombi. Their aggregation and consumption on injured endothelium leads to lowered platelet counts [68,69]. In the circulation, platelets are degranulated [70] with consequent elevated platelet-derived proteins such as platelet factor-4, β-thromboglobulin and P-selectin [71,72]. Shiga toxin binds to platelets via Gb3 and an alternative glycolipid receptor [73]. LPS binds to platelets via a toll-like receptor (TLR)4-CD62 receptor complex [74] and activates platelets. LPS derived from *E. coli* O157 was particularly potent in this respect [74]. Once activated, platelets further respond to Shiga toxin [75,76], may take up the toxin and exhibit excessive fibrinogen binding, enhancing their thrombotic potential. Platelet activation induced by Shiga toxin may be further exacerbated by toxin-mediated endothelial cell damage, exposing the subendothelium with release of von Willebrand factor, fibrinogen and collagen [68,69], as well as complement deposition. Furthermore, complement deposits on platelet and platelet–leukocyte aggregates in response to stimulation with Shiga toxin and *E. coli* O157 LPS [77]. Studies have shown that complement deposition on platelets initiates thrombin-mediated aggregation [78]. Moreover, once thrombin is formed [79], it can further propagate complement system activation [80,81] enhancing the inflammatory activity at the interface of platelets and the endothelium [82]. Once activated, platelets may contribute to the inflammatory state by the release of potent chemokines, as previously reviewed [9,69].

Patients with HUS exhibit acute hemolysis with fragmented red blood cells [1]. Hemolysis may be caused by the mechanical breakdown of red blood cells within occluded blood vessels, but complement activation on red blood cells may also contribute to this process, since it is known that complement deposition on red blood cells induces hemolysis [83]. Patients with HUS exhibit C3 deposition on red blood cells [19], suggesting that complement is activated on blood cell surfaces. Shiga toxin binds to the Gb3 receptor on red blood cells, also known as the P^k^ antigen of the P1Pk blood group system [20,84]. In vitro experiments demonstrated that Shiga toxin induced hemolysis by activating complement on human red blood cells in the presence of plasma, an effect abrogated by complement inactivation or by addition of the terminal complement pathway inhibitor eculizumab, directed against C5 [19].

Taken together, Shiga toxin is capable of binding to neutrophils, monocytes, platelets and red blood cells and thus it may be transported on, or within, blood cells in the circulation. Blood cells are activated thus potentiating the inflammatory and thrombotic process occurring during HUS. Furthermore, the toxin may thereby reach its target organs [85], mainly the kidney and the brain, although this mechanism of transfer does not fully clarify how the toxin is released from blood cells and taken up by recipient cells [86].

### 2.4. Thrombus Formation During HUS

Shiga toxin binds to endothelial and epithelial cells expressing the Gb3 receptor, such as the glomerular endothelium and the tubular epithelium in the human renal cortex [52,87]. Interestingly, cytokine release and exposure to bacterial LPS enhances Gb3 expression and augments toxin binding [88,89]. The mechanisms contributing to thrombus formation are multifactorial involving toxin-mediated endothelial cell damage [90], platelet activation [74,76], the formation of platelet-monocyte aggregates expressing tissue factor [18] as well as the release of inflammatory mediators from monocytes and endothelial cells that further activate platelets [66,91]. In addition, activation of coagulation and impaired fibrinolysis occur before HUS develops [79] promoting thrombus stabilization during HUS, after which fibrinolysis is enhanced [92,93,94]. Of note, the fibrinolytic system is activated in the murine kidney both in the endothelium and in tubular epithelial cells [95] but Shiga toxin may actually lower production of fibrinolytic parameters by inhibiting protein synthesis [94] and thereby tip the balance towards enhanced thrombus formation.

### 2.5. Shiga Toxin Induces the Release of Blood Cell-Derived Microvesicles

Binding of Shiga toxin to circulating blood cells may initiate cell activation and the release of blood cell-derived microvesicles, particularly in the presence of LPS [18,77]. During the acute phase of HUS, levels of circulating microvesicles are elevated [18,19,21,96] and Shiga toxin was detected within blood cell-derived microvesicles originating from neutrophils, monocytes, platelets and red blood cells [21]. In the following section, we will describe extracellular vesicles, their formation, shedding and uptake. We will also describe the function of microvesicles in normal cellular interactions and contribution to pathological processes, in particular during infectious diseases. Thereafter, we will focus on their contribution to HUS.

## 3. Characteristics of Extracellular Vesicles

**Figure 1:**
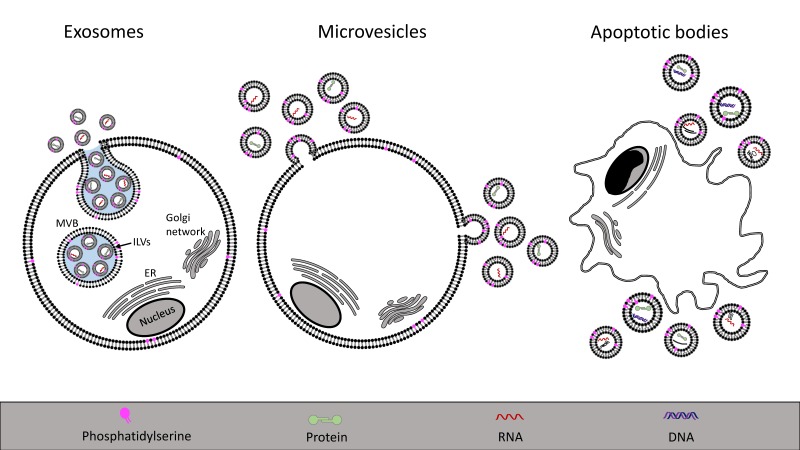
Schematic presentation of the mechanisms of extracellular vesicle release. Exosomes, shown on the left, are derived from multivesicular bodies (MVB) that are late endosomes carrying intraluminal vesicles (ILVs). MVBs fuse with the plasma membrane to release the ILVs that are termed exosomes once shed into the extracellular space. Microvesicles, shown in the middle panel, originate from a direct budding of the plasma membrane. Apoptotic bodies, depicted on the right, are released during apoptosis and cellular degradation. ER: endoplasmic reticulum.

Extracellular vesicles are membranous particles, released from cells, that are characterized based on their cell of origin and their size (Figure 1). Exosomes (30–100 nm in diameter) are formed by the release of the contents of intracellular endosomal multivesicular bodies containing intraluminal vesicles. Once extruded, these vesicles are termed exosomes [97]. Microvesicles (100–1000 nm) are released from cells by direct budding of the plasma membrane, while apoptotic bodies (1–5 μm) are released by the breakdown of cells during programmed cell death [98]. The latter are distinctly different from other vesicles because they contain larger cellular degradation products, such as organelles. The properties of extracellular vesicles are summarized in Table 1. Detection methods include flow cytometry, nanoparticle tracking and transmission electron microscopy, among others, as well as proteomic analysis of vesicular contents, as reviewed elsewhere [99,100,101,102]. Extracellular vesicles can be differentiated based on their mechanism of secretion and based on cellular markers [29,103], often allowing to determine the parent cell from which the vesicles were released. Web-based databases are available in which data regarding vesicle content and properties is summarized (see Vesiclepedia, ExoCarta or EVpedia, online).

### 3.1. Exosomes

Intracellular multivesicular bodies are late endosomes containing intraluminal vesicles. When they dock onto the plasma membrane and fuse, the vesicles are released as exosomes into the extracellular space [104]. The formation of intraluminal vesicles and their release as exosomes is mainly regulated by two pathways, either via the endosomal sorting complex required for transport (ESCRT) machinery [105,106] or by an ESCRT-independent process (including tetraspanins and ceramide generation) [107,108]. Exosomes are characteristically different from other vesicle populations due to their endosomal origin with a unique composition of lipids and proteins. Compared to the parent cell, exosomes may be enriched in cholesterol, glycosphingolipids, sphingomyelin and phosphatidylserine [109]. Proteins enriched in exosomes include tetraspanins [110], heat shock proteins, component of the ESCRT family [111] and MHC class I [112]. While these proteins are generally enriched in exosomal populations, other proteins may be specifically enriched in exosomes dependent on the cell of origin [111,113]. Comprehensive reviews regarding exosomal formation and their biological functions have been published elsewhere [103,104,114], and herein we will focus on microvesicles.

### 3.2. Microvesicles

Microvesicles are shed directly from the plasma membrane together with contents of the parent cell. The content may include proteins (cytokines, chemokines, enzymes, receptors) [32,115], RNAs (mRNAs and non-coding RNAs, particularly miRNAs) [23] and lipids [116]. The content will vary depending on the cell of origin and reflect the biological status of the cell and degree of activation [117]. The structure of microvesicles protects bioactive materials and enables the transfer of such substances from one cell to another as a means of cell-to-cell communication [29,118,119]. Microvesicles can be taken up by neighboring or distant cells and have the potential to phenotypically alter the recipient cell [120,121], as elaborated on below. Another important function of microvesicles is to discard the parent cell of unwanted substances and thus preserve cellular integrity [122,123,124].

#### 3.2.1. Microvesicle Formation

The underlying mechanism of microvesicle formation involves numerous cellular events leading to local budding of the plasma membrane, followed by a fission event whereby the vesicle is pinched off and released into the extracellular space [125]. Shedding of microvesicles occurs spontaneously in resting cells [23], and is increased during cell activation in response to various stimuli such as hypoxia, oxidative stress, shear stress, pro-inflammatory mediators, cell damage or ligand-binding [126,127,128]. A common feature during activation is an increase in the cytosolic calcium level, which promotes microvesicle release [129]. Increasing evidence suggests that the inner content as well as the surface of microvesicles are selectively packaged, rather than randomly, by a controlled mechanism of cargo trafficking within microvesicles, as reviewed [121]. The regulatory protein, ADP-ribosylation factor 6, selectively recruits protein cargo onto microvesicles and has been shown to be an important component of this process [130]. The exact mechanisms by which proteins, lipids and RNAs are targeted into microvesicles remain to be elucidated. The process is affected by external stimuli triggering vesicle release, as well as by the cellular environment [126]. The same cell may thus release microvesicles with varied content [131]. Interestingly, microvesicles may incorporate membranous contents at a higher density than the parent cell due to enrichment in lipid rafts [132], thereby enhancing the procoagulant potency of platelet-derived microvesicles [133].

In resting cells, plasma membrane lipids are arranged in an asymmetric pattern whereby the outer leaflet contains phosphatidylcholine and sphingomyelin, and the inner membrane leaflet is enriched with aminophospholipids (phosphatidylserine and phosphatidylethanolamine) [134,135]. Phospholipid asymmetry is controlled by specific enzymes such as flippase (transfers phosphatidylserine to the inner leaflet), floppase (controls outward phospholipid translocation), and enzymes with scramblase activity (transport lipids in a bidirectional Ca^++^-dependent manner) [136,137]. During cell activation, concurrent with a rise in the intracellular calcium level, floppase and scramblase activities are activated, while flippase is inactivated leading to disruption of lipid asymmetry with exposure of phosphatidylserine on the outer leaflet. The increased calcium level also enhances the activity of cytosolic enzymes such as calpain, involved in cytoskeleton changes that facilitate microvesicle shedding [138]. Microvesicle membranes are characterized by a loss of lipid asymmetry, in comparison to the parent cell, and may expose phosphatidylserine, although this is not a feature of all microvesicles [126]. The mechanism associated with the release of phosphatidylserine-negative microvesicles is poorly understood. A possible mechanism involving cellular proteins able to alter the membrane curvature has been described [139].

#### 3.2.2. Microvesicle Uptake

Uptake of microvesicles by recipient cells can be mediated by various endocytic pathways or fusion with the plasma membrane. Endocytosis may be facilitated by receptor-ligand interactions. Uptake may involve clathrin-dependent or -independent endocytosis, caveolin-dependent endocytosis, macropinocytosis, phagocytosis or lipid raft-mediated uptake, as reviewed [140]. Fusion of microvesicles with the membrane of the target cell is a highly dynamic process, resembling the process utilized by retroviruses, involving high affinity binding to the target cell, lipid reorganization and restructuring of proteins [141]. The fusion event enables the bioactive vesicular content to be inserted into the cytosol of the target cell [142]. Several factors have been shown to affect fusion; these include an acidic environment, the degree of ligand-receptor binding and the lipid composition of the microvesicle [143].

The uptake of a microvesicle may depend on the presence of receptors and/or proteins on the microvesicle surface and their interaction with counterparts on the recipient cell [140,144]. These interactions may lead to the formation of a microvesicle–cell complex, thereby coating the cell and possibly initiating intracellular signaling events due to receptor-ligand interactions. The microvesicle may be taken up by the cell and release its content into the cell [119]. Ligands specifically enriched on microvesicles such as glycoproteins (integrins, selectins and tissue factor) and phospholipids may favor cell-specific uptake [145,146,147,148]. For example, platelet-derived microvesicles transfer tissue factor to monocytes but not to neutrophils [149], demonstrating uptake by a defined cell type.

#### 3.2.3. The role of Microvesicles in Intercellular Communication

The diversity of bioactive materials covering the surface of microvesicles and contained within microvesicles may affect the phenotype of the recipient cell by transfer of nucleic acids, proteins, receptors and lipids. RNAs may be translated in the target cell activating or silencing certain properties [150,151,152]. The physiological or pathological consequences may result in induction of angiogenesis, thrombosis, altered hemostasis, immune modulation, invasive potential, matrix alteration and tissue regeneration, as recently reviewed [32]. The transfer of functionally active receptors may activate pro-inflammatory or pro-thrombotic signaling pathways as well as proliferative capacity in the recipient cell, as has been exemplified regarding chemokine receptors [153,154], epidermal growth factor receptor [155], kinin B1 receptor [156] and platelet receptors [157]. The transfer of the platelet receptor GPIIb/IIIa to neutrophils will induce neutrophil binding to the endothelium via fibronectin and thereby have a pro-inflammatory effect [158]. Cancer cell-derived microvesicles have been shown to bear epidermal growth factor receptor, that could be transferred to neighboring cells and influence cell morphology and growth capacity [120]. The invasive capacity has been demonstrated in microvesicles derived from cancer cells which are enriched in metalloproteinases capable of breaking down extracellular matrix to promote tumor growth [159].

Microvesicles derived from blood and endothelial cells may induce an inflammatory response. As mentioned above, microvesicles can transfer chemokine receptors [153]. Furthermore, microvesicles from platelets or from endothelial cells can induce monocyte [160] or neutrophil chemotaxis [161]. The recruited monocytes will deposit on endothelial cells [162]. Likewise, platelet-derived microvesicles can recruit CD34-positive hematopoietic cells [157]. The immunomodulatory effects of microvesicles also encompass the transfer of anti-inflammatory mediators (RNAs or proteins), particularly studied in microvesicles derived from stem cells [163] or neutrophils [164], potentially contributing to a beneficial effect during tissue regeneration.

Importantly, microvesicles may possess pro-thrombotic potential. The exposure of phosphatidylserine on the outer leaflet of microvesicles generates a negatively-charged surface promoting binding of prothrombin, factor Va and factor Xa [165]. When scramblase activity is defective, such as in Scott syndrome, the number of phosphatidylserine-positive microvesicles is reduced and coagulation is impaired resulting in a bleeding disorder [166,167]. Phosphatidylserine exposure on monocytic microvesicles was shown to differ depending on the stimulus inducing microvesicle release suggesting that environmental factors can affect the composition of microvesicles and their pro-thrombotic properties [128]. In addition, certain microvesicles, mainly from monocytes and platelets, carry tissue factor on their surface contributing to thrombus formation [18,146,168,169]. Monocyte-derived microvesicles transfer tissue factor to platelets [170]. Red blood cell-derived microvesicles are also thrombogenic and may induce thrombin generation in the absence of tissue factor by activation of the intrinsic coagulation pathway on their surfaces [171]. Similarly, platelet-derived microvesicles containing arachidonic acid metabolized to thromboxane A2 induce platelet aggregation [116].

## 4. Microvesicles in Infectious Diseases

Infections, and sepsis in particular, are accompanied by a pro-inflammatory and pro-thrombotic host response. The properties of blood cell-derived microvesicles can contribute to these effects and to multi-organ failure. Patients with sepsis have high levels of platelet- and leukocyte-derived microvesicles [172,173,174,175]. Blood cell-derived microvesicles were tissue factor-positive in patients with meningococcal sepsis [172] and febrile urinary tract infection [176]. Patients with disseminated intravascular coagulopathy had elevated endothelial and leukocyte microvesicles [177]. An enhanced inflammatory response during sepsis, as reflected by microvesicle levels, may, however, predict a more favorable outcome [178], and circulatory microvesicle levels correlated negatively with the degree of acute kidney injury during sepsis, although this may actually reflect the degree of microvesicle deposition in damaged tissue [174].

When microvesicles released during sepsis were injected into mice they enhanced vascular reactivity and thromboxane A2 production [175]. Likewise, microvesicles derived from rats with peritonitis induced an inflammatory response when injected into healthy rats [179]. Thus, microvesicles may have damaging effects during sepsis [180,181]. Beneficial effects may also prevail as neutrophil-derived microvesicles, that were elevated during bacteremia, exhibited anti-microbial effects [182]. Similar effects were also demonstrated as blood cell-derived microvesicles prevented in vivo spreading of *Streptococcus pyogenes* [183].

### Microvesicles May Transfer Infectious Agents or Their Virulence Factors

Bacteria and viruses have developed ways of utilizing extracellular vesicles for the transfer of their antigens and virulence factors. For example, *Mycobacterium tuberculosis* infected macrophages release microvesicles containing *M. tuberculosis* antigen in complex with MHC-II that can induce an antimicrobial T-cell response [184]. HIV budding from cells can utilize components of the ESCRT pathway and yet be shed directly from the cell membrane [185,186] suggesting that viral dissemination may occur via extracellular vesicles [187] possibly utilizing phosphatidylserine receptors [188]. Other viruses, such as herpes viruses, also utilize extracellular vesicles to transfer viral RNAs and proteins from cell to cell [189,190].

Pathogens can thereby exploit the microvesicle transport system for their own benefit to evade host response by covering themselves with the host membrane within a vesicle. The vesicle containing pathogen virulence factors is taken up by recipient cells thereby affecting target cells. Interestingly, pathogens can also release their own vesicles. Protozoan parasites, such as *Trypanosoma cruzi*, release vesicles capable of interacting with host cells [191,192]. Similarly, mycobacterium may release vesicles capable of inducing an inflammatory response in the host [193]. Outer membrane vesicles are released from gram-negative bacteria, such as EHEC, and may transfer toxins, such as Shiga toxin, into intestinal epithelial cells as well as renal or brain endothelial cells [194]. The major difference between pathogen-derived vesicles [194] and host blood cell-derived microvesicles [21], both containing virulence factors, such as Shiga toxin, is that the latter, host-derived extracellular vesicles, will be recognized as “self” and thereby avoid attack by the immune system whereas pathogen-derived vesicles are “non-self” and subject to immune attack.

## 5. Shiga Toxin-Induced Microvesicles in Laboratory Models

Shiga toxin binds to neutrophils, monocytes, platelets and red blood cells [20,22,64,65,73]. These blood cells are resistant to the cytotoxic effects of the toxin, in part due to minimal protein synthesis in platelets and red blood cells. The cells may instead become activated and release microvesicles [18,19,66,76,77]. In vitro experiments have shown that Shiga toxin induces the release of microvesicles from human monocytes, platelets [18,77] and red blood cells [19]. Co-stimulation with Shiga toxin and *E. coli* O157-LPS enhanced microvesicle release from platelets and leukocytes compared to each stimulant alone [18]. Microvesicles shed from platelets and monocytes carry tissue factor and phosphatidylserine [18], as well as complement C3 and C9 [77]. C5b-9 was also detected on microvesicles from red blood cells which also exposed phosphatidylserine after stimulation with Shiga toxin [19]. The toxin itself may be incorporated in blood cell-derived microvesicles originating from neutrophils, monocytes, platelets and red blood cells [21]. Permeabilization of microvesicles was required to detect Shiga toxin, suggesting that the toxin was mainly localized within the microvesicles and not on their outer membrane [21].

**Figure 2:**
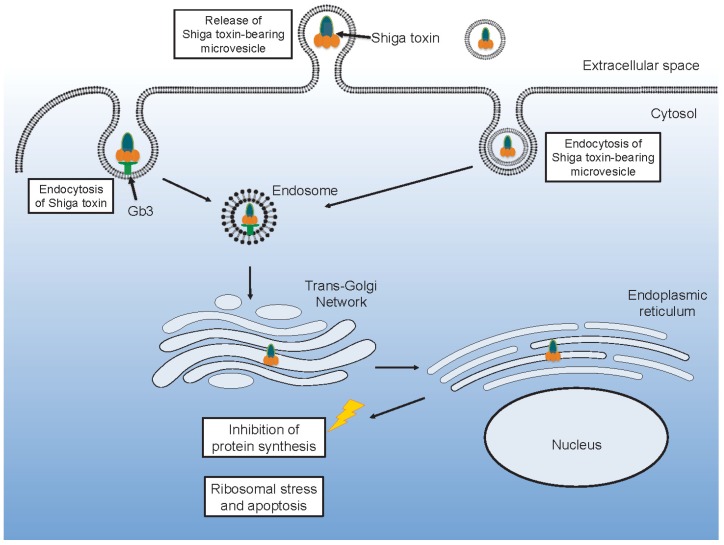
Shiga toxin uptake and intracellular routing. Shiga toxin may be taken up directly after binding to its glycolipid receptor Gb3. Alternatively, the toxin may be endocytosed within a microvesicle. Either way the toxin will undergo retrograde transport via the Golgi to the endoplasmic reticulum and the ribosomes where it may inhibit protein synthesis or induce ribosomal stress and apoptosis.

Toxin within microvesicles can be transferred to target organ cells and be taken up after endocytosis of the entire microvesicle [21] (Figure 2). In a mouse model of EHEC infection [62], blood cell-derived microvesicles carrying Shiga toxin were demonstrated to be taken up by renal glomerular endothelial cells and tubular cells [21]. One notable aspect of this finding is that, in contrast to the human glomerulus, mouse glomerular endothelial cells lack the toxin Gb3 receptor [87]. Thus, it is possible that this process is Gb3- or toxin receptor-dependent up until the toxin binds to its receptor on blood cells and is internalized in these cells. After the toxin is shed from the blood cells, within microvesicles, the toxin-containing microvesicles may be taken up even by cells that lack the Gb3 [1].

## 6. Microvesicles in the Pathogenesis of Hemolytic Uremic Syndrome

**Figure 3:**
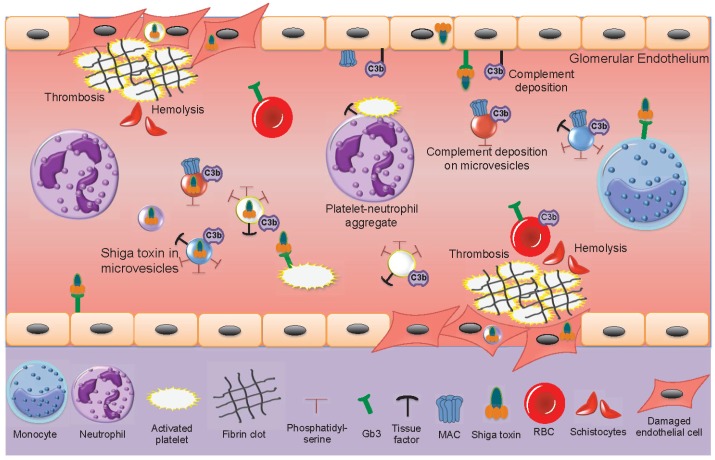
Shiga toxin-induced activation of blood cells and damage to the glomerular endothelium. Within the bloodstream Shiga toxin can bind to monocytes, neutrophils, activated platelets and red blood cells. Aggregates are formed between platelets and leukocytes (depicted in the figure between a neutrophil and a platelet). Microvesicles are shed from blood cells (neutrophils, monocytes, platelets and red blood cells). Some of these contain the toxin. All these microvesicles may expose phosphatidylserine. Microvesicles from platelets and monocytes expose tissue factor. Complement may deposit on platelet, monocyte and red blood cell-derived microvesicles, as well as on the glomerular endothelium. Shiga toxin binds to glomerular endothelial cells via the Gb3 glycolipid receptor or undergoes endocytosis within blood cell-derived microvesicles. Shiga toxin-induced cytotoxicity and complement deposition lead to glomerular endothelial cell damage. Thrombosis is induced by toxin-mediated endothelial cell damage, platelet activation and the pro-thrombotic effects of microvesicles. Hemolysis is induced by mechanical breakdown of red blood cells in occluded capillaries as well as by complement deposition on red blood cells. Fragmented red blood cells are termed schistocytes. Gb3: globotriaosylceramide, MAC: membrane attack complex (C5b-9), RBC: red blood cell.

During EHEC-associated HUS, only minimal amounts of freely circulating Shiga toxin have been found in patient samples and Shiga toxin is mainly found bound to blood cells [15,18]. In acute phase HUS patients, the number of circulating blood cell-derived microvesicles is elevated [18,19,21,96], decreasing after recovery. Shiga toxin circulates within microvesicles originating from neutrophils, monocytes, platelets and red blood cells [21]. Shiga toxin-containing microvesicles from blood cells were further demonstrated to be taken up by glomerular endothelial cells in a patient sample [21]. Kidney cells, including glomerular endothelial cells, mesangial cells, podocytes and tubular cells are highly sensitive to Shiga toxin [52,195,196]. The effects of Shiga toxin and blood cell-derived microvesicles in the circulation of acute phase HUS patients are described in Figure 3.

The main features of HUS are thrombocytopenia due to platelet consumption in microthrombotic lesions on the damaged endothelium, hemolysis with fragmented red blood cells and renal failure. Microvesicles may contribute to and partake in each of these processes. The pro-thrombotic properties of microvesicles, bearing tissue factor and exposing phosphatidylserine on their outer leaflet, as described above, and reviewed [32], may contribute to thrombin generation and platelet activation on damaged microvasculature. Complement deposition on red blood cells will induce hemolysis and the release of complement-coated red blood cell-derived microvesicles during HUS [19] suggesting that hemolysis is induced not only by mechanical fragmentation of red blood cells in occluded blood vessels but also by complement activation. The pro-thrombotic effect may be further enhanced by circulating red blood cell-derived microvesicles capable of activating the intrinsic coagulation pathway [171].

The presence of complement C3 and C9 on platelet and monocyte-derived microvesicles in HUS patients [77] would reflect complement activation on the parent cells and could also contribute to the thrombotic process as complement activation on platelet membranes may promote their activation [197]. During HUS complement is deposited on the injured vascular wall [198], on platelets and microvesicles derived thereof [77], suggesting that complement contributes to the tissue injury during HUS [82]. Indeed, mouse models of EHEC infection or Shiga toxin injection in which the alternative, lectin or terminal complement pathways were blocked, exhibited reduced renal injury [198,199,200].

Shiga toxin can be transferred from the bloodstream to the kidney within blood cell-derived microvesicles and taken up in glomerular endothelial cells or peritubular capillary endothelial cells [21]. This mechanism could explain how the toxin enters target organ cells after circulating within activated blood cells that release microvesicles. In a mouse model, toxin-positive microvesicles were released from blood cells during the early stages of infection before symptoms develop [21]. They were further taken up by renal cells before clinical signs of disease were evident. Microvesicles were demonstrated to release their content of Shiga toxin within 12 h of entering the cell and the toxin reached ribosomes within 24 h, inhibiting protein synthesis, thus providing evidence that toxin within microvesiscles retains its cytotoxicity. An interesting finding was that not all microvesicles emptied their contents after endocytosis, certain microvesicles transcytosed the recipient cells, migrated through the corresponding glomerular or tubular basement membranes and passed into podocytes or tubular cells, respectively [21]. The capacity of microvesicles to navigate through cells and basement membranes could be secondary to tissue injury during HUS or possibly the toxic content of the microvesicles.

## 7. Conclusions

During Shiga toxin-associated HUS, microvesicles are released from blood cells capable of transferring the toxin to target organ cells inducing renal cell death and promoting thrombosis. Red blood cell-derived microvesicles are also involved in the hemolytic process. Microvesicles are thereby involved in all aspects of HUS.

Microvesicles transfer an array of nucleic acids, proteins and lipids, and are thus a potent mechanism for intercellular communication, which may have detrimental effects during infection and inflammation, but may also have beneficial effects in tissue regeneration. Furthermore, the shedding of microvesicles carrying unwanted cellular components may maintain cell integrity after a trigger of cell activation. Therefore, future studies should address the importance of microvesicles and possible effects of blocking their release in Shiga toxin-mediated infection and kidney damage.

## Figures and Tables

**Table 1:** Classification of extracellular vesicles.

Extracellular Vesicle	Origin	Size	Content	References
Exosome	Intraluminal vesicle within multivesicular bodies	30–100 nm	mRNA, miRNA, proteins, lipids	[97]
Microvesicle	Shedding from the plasma membrane with cellular content	100–1000 nm	mRNA, miRNA, proteins, lipids	[29]
Apoptotic body	Cellular breakdown and shrinkage during apoptosis	1–5 μm	Organelles, proteins, DNA, RNAs, lipids	[97]
